# Complete Genome Sequence of *Kluyveromyces lactis* Strain GG799, a Common Yeast Host for Heterologous Protein Expression

**DOI:** 10.1128/genomeA.00623-17

**Published:** 2017-07-27

**Authors:** Léa Chuzel, Mehul B. Ganatra, Kelly M. Schermerhorn, Andrew F. Gardner, Brian P. Anton, Christopher H. Taron

**Affiliations:** New England Biolabs, Ipswich, Massachusetts, USA

## Abstract

We report the genome sequence of the dairy yeast *Kluyveromyces lactis* strain GG799 obtained using the Pacific Biosciences RS II platform. *K. lactis* strain GG799 is a common host for the expression of proteins at both laboratory and industrial scales.

## GENOME ANNOUNCEMENT

*Kluyveromyces lactis* is an ascomycetous yeast from the family *Saccharomycetaceae* that has a long history in research and biotechnology. *K. lactis* produces lactase, an enzyme that breaks down milk sugar (lactose). It is a major source of lactase for dairy industry processes. Additionally, *K. lactis* is a popular host for protein expression. Over 100 proteins have been expressed in *K. lactis* (1, 2), including chymosin that is produced at industrial scale (3). Here we report the complete genome sequence of *K. lactis* strain GG799, a common host strain for protein expression.

A 10-kb SMRTbell library was prepared from genomic DNA extracted from *K. lactis* GG799 cells with a blood and cell culture midikit (Qiagen), size selected with a BluePippin (Sage Science) for fragments over 8 kb, and sequenced with an RS II using P6 chemistry, 6 single-molecule real-time (SMRT) cells, and 360-min movies (Pacific Biosciences). Reads from 3 SMRT cells were assembled *de novo* using the RS_HGAP_Assembly.2 protocol, generating 70 contigs with 137× mean coverage.

The haploid genome of GG799 comprises six chromosomes (A through F). Five of six chromosomes (A, B, C, E, and F) were complete, and each was assembled into a single contig. Chromosome D, although complete, was split into two contigs at the ribosomal DNA (rDNA) tandem repeat region. Our reconstruction of chromosome D included two 8.7-kb rDNA repeat copies, but we estimated from sequence coverage that ~35 repeats exist. Of the additional 63 *de novo* contigs not described above, 28 were from mitochondrial DNA (mtDNA) and the remainder were redundant (5 from subtelomeric chromosomal repeat regions and 30 from the rDNA repeat region of chromosome D). The six complete chromosomes and mtDNA were error corrected using data from all six SMRT cells, with mean coverages of ~300× and ~1,900×, respectively. The total length of the GG799 genome was 10.7 Mb, with a GC content of 38.7% (including mtDNA).

The assembled contigs were mapped to the previously sequenced *K. lactis* strain CLIB210 (4, 5). The *K. lactis* strains GG799 and CLIB210 have highly similar genome organization. The only major difference is at the mating-type locus (MAT) on chromosome C. Strain GG799 harbors an MATα allele 250 kb into the 5′ region of chromosome C, whereas CLIB210 contains an MAT**a** allele at the same position. This difference reflects the opposite mating phenotypes of both strains (6). Interestingly, GG799 lacks an extra copy of HMLα at the 5′ end of chromosome C that is present in CLIB210.

Both strains also have subtelomeric regions comprising many duplicated sequences, but their organization is not identical, a finding that is consistent with the known plasticity of these fast-evolving regions (7).

This study provides a second *K. lactis* genome sequence that will permit further comparative insights into the evolution of *Kluyveromyces* yeasts. Additionally, this work will facilitate more informed engineering of *K. lactis* GG799 for improved heterologous protein expression.

### Accession number(s).

The complete genome sequence has been deposited in GenBank under the accession no. CP021239 to CP021245. The version described in this paper is the first version.
